# Spared corticospinal neurons activate an endogenous plasticity program after partial CNS injury

**DOI:** 10.64898/2026.09.08.750239

**Published:** 2026-09-14

**Authors:** Matias Murillo, Ciara F O’Brien, Noa Golan, Emma X Yin, Kristen J Brennand, William B J Cafferty

**Affiliations:** 1 Interdepartmental Neuroscience Program, Yale University School of Medicine, New Haven, CT 06511, USA.; 2 Department of Neurology, Yale University School of Medicine, New Haven, CT 06511, USA.; 3 Department of Psychiatry, Yale University School of Medicine, New Haven, CT 06511, USA.; 4 Present address: School of Biological Sciences, 1 Bungtown Road, Cold Spring Harbor Laboratory, Cold Spring Harbor, NY, USA.; 5 Senior authors; 6 Lead contact

## Abstract

Functional recovery after incomplete spinal cord injury depends substantially on the capacity of anatomically spared neurons to remodel their connections, yet the molecular programs underlying this endogenous plasticity remain poorly understood. To identify and validate these mechanisms, we combined retrograde labeling, unilateral corticospinal tract injury, spatial transcriptomics, and human stem cell-derived neurons. Injured corticospinal neurons exhibited widespread downregulation with remaining activated pathways dominated by stress, cell death, and degenerative programs. In contrast, spared corticospinal neurons activated a coordinated pro-plasticity program characterized by metabolic, immune, and cytoskeletal remodeling together with selective suppression of growth-inhibitory signaling. Network and drug perturbation analyses identified *ARHGEF12* suppression and vorinostat treatment as complementary target- and state-based strategies to enhance neurite regeneration in human neurons. Together, these findings define key components of endogenous plasticity and provide a framework for discovering therapeutic targets for neural repair.

## Introduction

Stroke, traumatic brain injury, and spinal cord injury are leading causes of long-term disability because the adult central nervous system (CNS) has a limited capacity for axon regeneration^1^. Nevertheless, incomplete injuries permit a modest degree of spontaneous functional recovery that is largely attributed to remodeling of anatomically spared circuits^2–4^. Previous transcriptomic studies using single-nucleus RNA sequencing (snRNA-seq), translating ribosome affinity purification sequencing (TRAP-seq), and Patch-seq have identified transcriptional programs associated with axotomy, regeneration failure, and regenerative interventions^5–7^. These studies have provided important insight into the molecular response of injured corticospinal neurons (CSNs) following CNS injury. In contrast, comparatively little is known about the molecular programs that enable spared neurons to undergo adaptive plasticity and establish new functional connections. Defining these endogenous repair mechanisms could reveal therapeutic strategies that enhance recovery without requiring long-distance regeneration of injured axons.

We previously demonstrated that actively sprouting CSNs exhibit a distinct transcriptional program that can be leveraged to enhance corticospinal remodeling and functional recovery after injury^4^. However, the study was performed in *Ngr1* null mice and was limited to a small population of retrograde-labeled sprouting CSNs profiled at a single late post-injury time point using bulk RNA sequencing of laser-capture microdissected neurons. Consequently, it remains unknown whether anatomically spared CSNs more broadly engage a coordinated endogenous molecular response after injury and how that response differs from axotomy.

Recent advances in spatial transcriptomics now permit genome-wide interrogation of gene expression at high spatial resolution while preserving the anatomical architecture of the tissue^8,9^. Although we and others have previously profiled intact CSNs using fluorescence-activated cell sorting and single-cell RNA sequencing^10,11^, this approach requires prolonged tissue dissociation and cell isolation that can induce stress-associated transcriptional artifacts. These challenges are amplified following CNS injury, when axotomized CSNs are particularly vulnerable to tissue processing. Spatial transcriptomics enables rapid capture of mRNA directly from fresh frozen tissue, minimizing *ex vivo* perturbations while capturing both cytoplasmic and nuclear transcripts from CSNs within their native cortical microenvironment. This approach therefore provides an opportunity to directly compare the transcriptional states of injured and spared CSNs.

Functional validation and clinical translation of candidate therapeutic targets remain important challenges in CNS regeneration research. Despite substantial advances in preclinical studies with animal models, relatively few restorative therapies have successfully translated into clinical practice^12–14^. Although rodents and humans share substantial genetic homology, important differences in corticospinal tract (CST) anatomy, circuit organization, and neuronal development raise the possibility that mechanisms governing axon growth and repair are not fully conserved across species^15–17^. Human induced pluripotent stem cell (iPSC)-derived cortical neurons provide a scalable and genetically tractable platform for modeling neurite regeneration in a human genetic background. Integrating transcriptomic discovery *in vivo* with functional validation in human iPSC-derived neurons therefore establishes a complementary framework for identifying and prioritizing therapeutic strategies with greater translational potential.

Here, we combined retrograde labeling, unilateral CST injury and spatial transcriptomics to define the transcriptional programs of injured and spared CSNs. We identified a coordinated endogenous pro-plasticity signature in spared neurons that was distinct from the injury response and leveraged this signature to prioritize a genetic regulator, ARHGEF12, and a pharmacological intervention, vorinostat. We subsequently validated both as modulators of neurite regeneration in human iPSC-derived cortical neurons. Together, these findings define key components of endogenous plasticity in response to CNS injury and establish an integrative framework for discovering and validating therapeutic targets for neural repair.

## Results

### Integrated spatial transcriptomic strategy enables comparative profiling of injured and spared CSNs.

**Figure 1.**
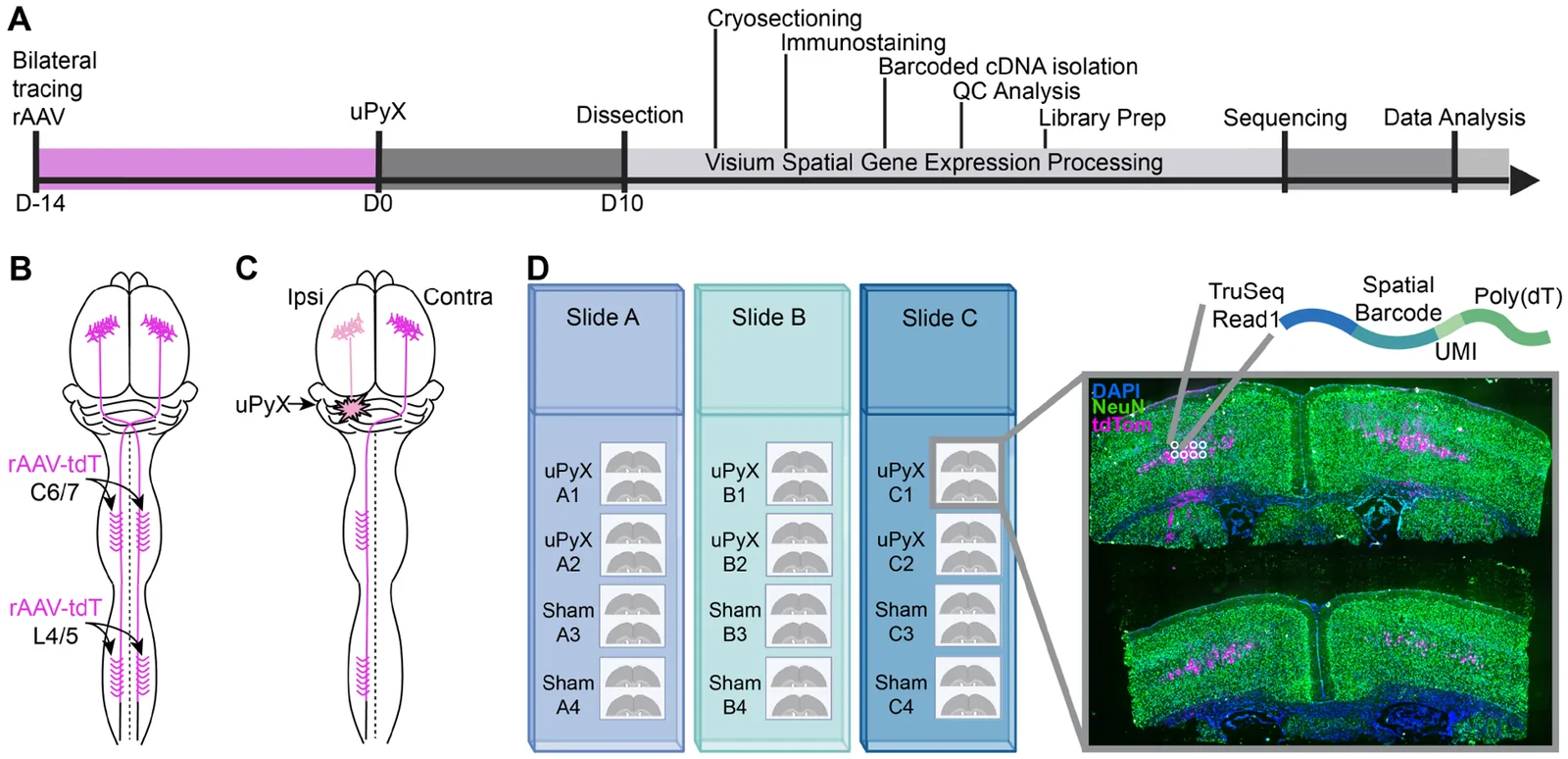
Integrated spatial transcriptomic strategy enable comparative profiling of injured and spared CSNs. **(A)** Experimental timeline. **(B)** Bilateral retrograde labeling for cervical and lumbar CSNs. **(C)** Injury model. Unilateral pyramidotomy (uPyX) selectively transects corticospinal tract axons originating from the ipsilateral hemisphere while preserving contralateral CSNs. **(D)** Distribution of uPyX and sham tissue sections across three Visium slides (Slides A–C). Inset: Representative M1 sections showing DAPI (blue), NeuN (green), and tdTomato-labeled CSNs (magenta) positioned over the Visium capture area. Inset illustrates Visium spots containing oligonucleotides with sequencing adaptor, spatial barcode, unique molecular identifier (UMI), and poly(dT) region.

To identify and compare the transcriptional states of injured and spared corticospinal neurons (CSNs), we designed an experimental strategy combining retrograde labeling, unilateral corticospinal tract injury (pyramidotomy uPyX) and spatial transcriptomics (Figure 1). CSNs were labeled via bilateral injections of AAVrg-CAG-tdTomato into the cervical or lumbar spinal cord two weeks prior to uPyX (Figures 1A, 1B). The uPyX selectively transects corticospinal tract (CST) axons at the level of the medullary pyramids, without damaging the cell bodies or local cortical circuitry^4^. Crucially, the unilateral nature of the injury specifically targets CSNs from the ipsilateral cortex while sparing the contralateral side (Figure 1C). Visium spatial RNA-sequencing was performed on coronal sections of primary motor cortex (M1) 10 days after injury (Figure 1A). Injured and spared CSNs within the same cortex enabled direct within-animal comparisons, while the sham animal sections served as baseline for intact gene expression and between-animal comparison. Histological assessment confirmed robust bilateral labeling of CSN populations and preserved tissue morphology (Figure 1D). This strategy enabled characterization of gene expression changes in injured and spared CSNs, within their native cortical environment.

### Anatomically-resolved gene expression maps were conserved across biological replicates.

**Figure 2.**
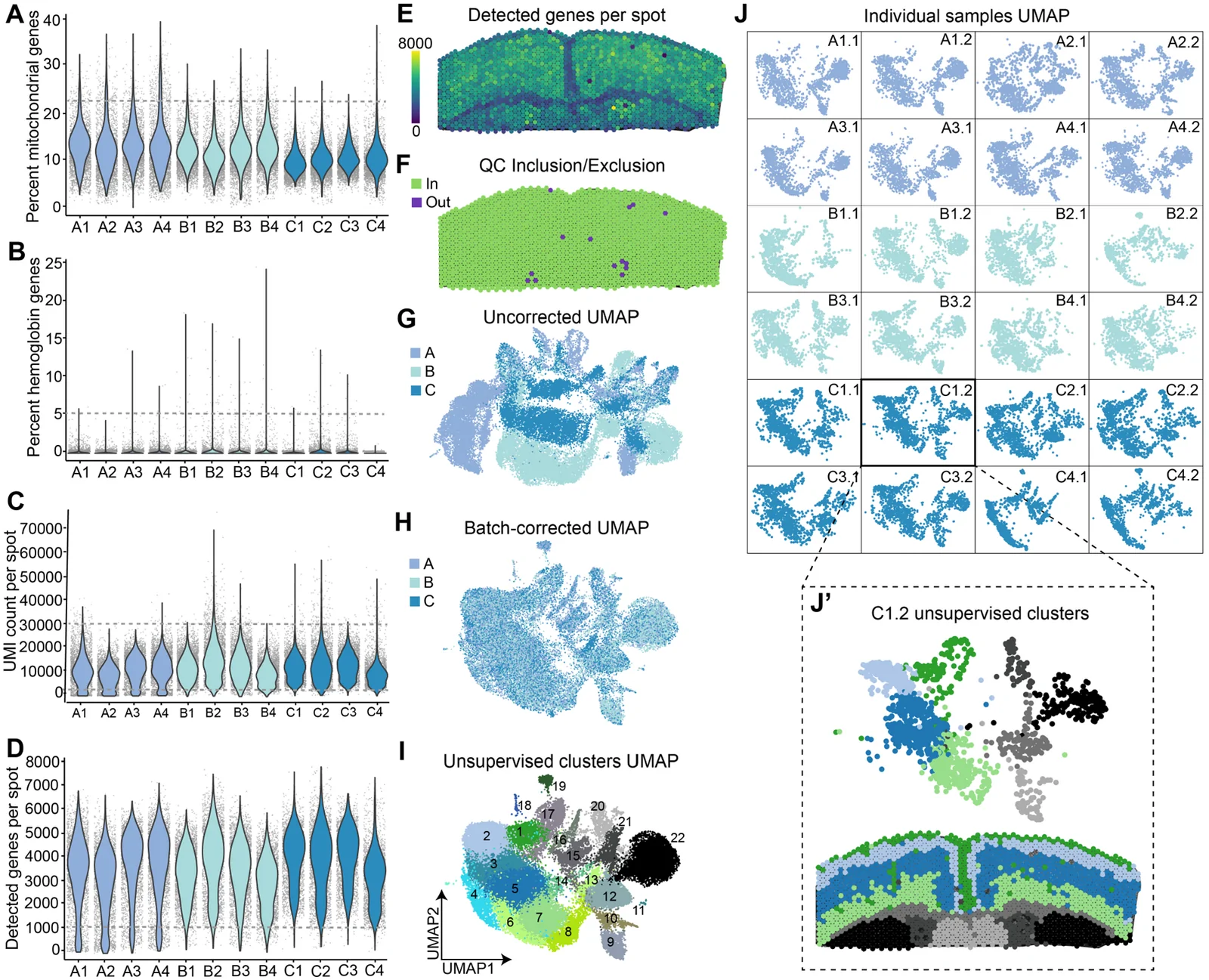
Anatomically-resolved gene expression maps were conserved across biological replicates. **(A–D)** Quality-control metrics across all Visium capture areas. **(A)** Percent mitochondrial reads, mean = 12.32%. **(B)** Percent hemoglobin reads, mean = 0.10%. **(C)** UMI counts per spot, mean = 11,230.24. **(D)** Detected genes per spot, mean = 3,583.04. Dashed lines represent cutoff thresholds. **(E)** Spatial distribution of detected genes across a representative tissue section. **(F)** Spots included (green) or excluded (purple) following quality-control filtering. Inclusion thresholds: < 22.5% mitochondrial counts, < 2.5% hemoglobin counts, > 1,000 genes, and between 1,000 and 30,000 UMIs. **(G)** UMAP before batch correction. **(H)** UMAP following Harmony batch correction. **(I)** Unsupervised Leiden clustering for all samples combined. **(J)** UMAP distribution across individual tissue sections. **(J′)** UMAP (top) and spatial localization (bottom) of unsupervised clusters for representative tissue section. Data are shown for n = 12 mice, represented by 24 tissue sections. Individual Visium spots are shown as individual data points.

Next, we assessed sequencing quality, library complexity and reproducibility (Figure 2). The low percentage of mitochondrial and hemoglobin reads per spot were consistent with preserved tissue quality and minimal blood contamination (Figures 2A, 2B). Conversely, the high number of UMIs and detected genes per spot confirmed robust capture of transcripts (Figures 2C, 2D). We mapped the detected gene counts per spot back to the tissue and found an expected pattern of high library complexity in cell-rich regions, such as the cortex, and reduced gene counts in the midline and corpus callosum (Figure 2E). Quality-control filtering resulted in minimal exclusion, with most spots per tissue section retained for downstream analyses. (Figure 2F). UMAP visualization of uncorrected data revealed separation partially driven by slide of origin (Figure 2G), which was effectively removed after batch-correction, resulting in coherent clustering across slides (Figure 2H). Unsupervised Leiden clustering on the corrected UMAP space identified 22 clusters (Figures 2I). Importantly, cluster identities and distribution within the shared UMAP were largely conserved across biological replicates (Figure 2J). Moreover, spatial mapping of clusters to the tissue revealed alignment to discrete anatomical regions including known cortical lamination patterns (Figure 2J’). This quality control and clustering pipeline demonstrated consistent sequencing performance and mapping of anatomically resolved gene expression patterns across biological replicates.

### Cell-type deconvolution identified cortical layer and CSN subpopulations.

**Figure 3.**
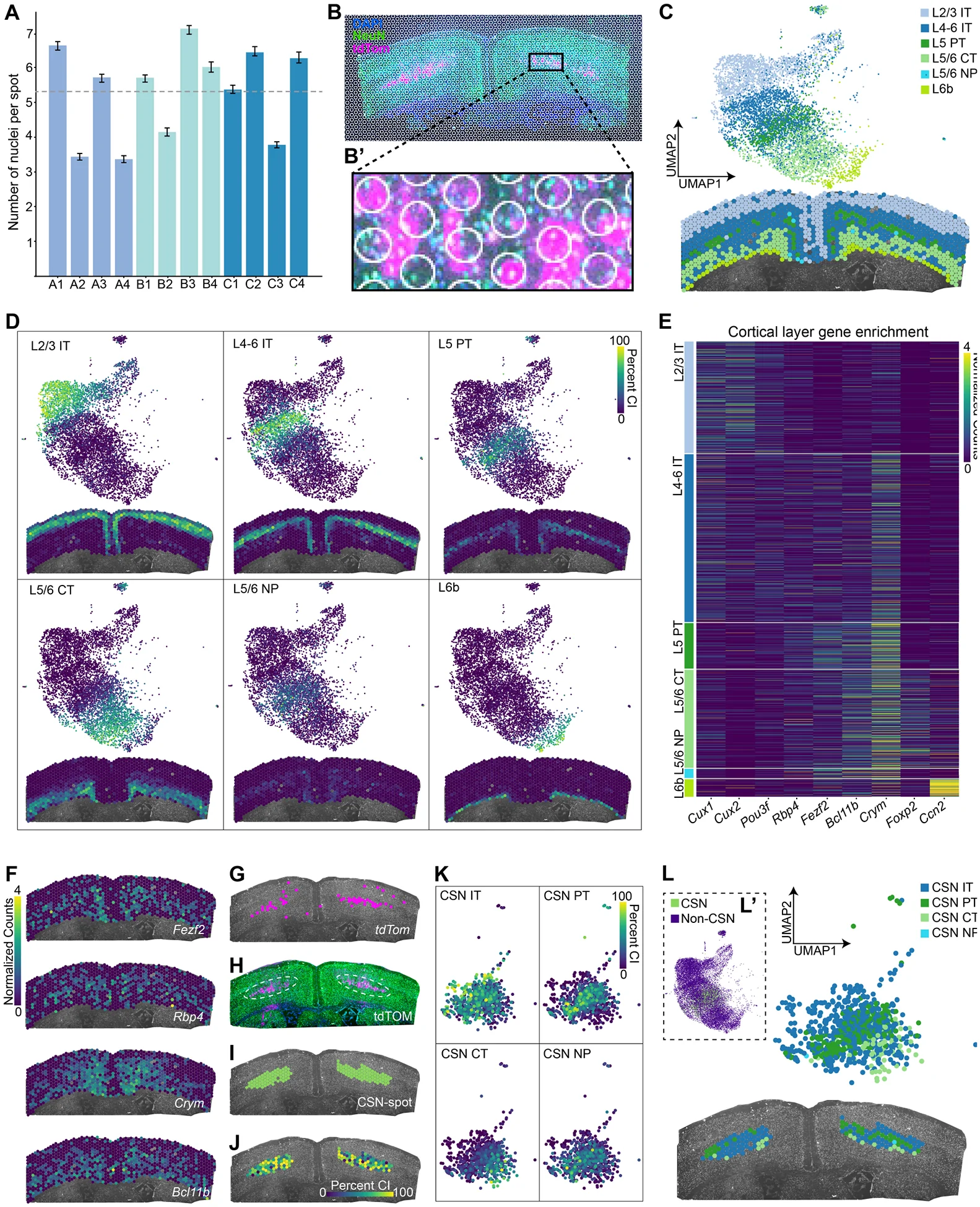
Cell-type deconvolution identified cortical layer and CSN subpopulations. **(A)** Number of nuclei per 55-μm Visium spot. Data represents mean ± SEM; n = 12 mice. Mean across mice = 5.32 (dashed line); SEM = 0.38. **(B)** Visium spot grid overlaid on cortical tissue. **(B′)** Higher-magnification view illustrating spatial resolution. **(C)** Cortical subpopulations identified by Stereoscope deconvolution using the Allen Institute M1 reference atlas. UMAP representation (top) and spatial distribution (bottom). IT, intratelencephalic; PT, pyramidal tract; CT, corticothalamic; NP, near-projecting. **(D)** Prediction confidence intervals (CI) for each inferred cortical population shown in UMAP space (top) and tissue space (bottom). **(E)** Heatmap of canonical cortical layer marker gene enrichment across predicted cortical populations. **(F)** Spatial expression of canonical layer 5 and CSN marker genes. **(G)** Spatial localization of tdTomato transcripts. **(H)** Immunofluorescence detection of tdTomato-labeled CSNs. **(I)** Regions of interest selected for downstream analyses. **(J)** Validation of selected CSN spots using a previously generated adult CSN single-cell RNA-seq reference dataset. **(K)** Deconvolution of CSN-enriched spots into subpopulations using the Allen Institute M1 reference atlas. Prediction confidence intervals (CI) shown in UMAP space. **(L)** Distribution of inferred CSN subtypes shown in UMAP space (top) and tissue space (bottom). **(L′)** Inset showing predicted CSN identities relative to all cortical spots.

To identify cell-type composition in M1 and isolate CSN-enriched spots for downstream analysis, we performed deconvolution aided by retrograde labeling and integration of single-cell RNA sequencing (scRNAseq) data (Figure 3). On average the 55μm spots contained 3–7 nuclei (Figure 3A). By overlaying the spot grid over the tissue, we identified the expected limitations imposed by the discontinuous distribution and spherical shape of the spots; individual cells may be missed entirely or captured only partially (Figures 3B, 3B’). These observations underscore the necessity of deconvolution to infer the most prominent cell type per spot. Each spot was assigned a probabilistic cell-type identity based on the Allen Institute for Brain Science’s scRNAseq reference atlas^18^. The neuronal cell types corresponded to canonical M1 neuronal subtypes, including L2/3 intratelencephalic (IT), L4–6 IT, L5 pyramidal tract (PT), L5/6 corticothalamic (CT), L5/6 near-projecting (NP), and L6b populations. The spatial distribution of these inferred identities recapitulated known laminar organization of M1 (Figure 3C). Confidence intervals for each spot over the tissue and in UMAP space confirmed accurate assignments to discrete cortical layers (Figure 3D). Deconvolution-derived identities were validated by expression of established layer-specific marker genes: upper-layer markers *Cux1/2*, layer V markers *Fezf2, Bcl11b, Crym*, and deep-layer 6b marker *Ccn2* (Figure 3E). Although, CSN-associated markers such as *Fezf2*, *Bcl11b*, *Crym*, and *Rbp4*^11,19^ were broadly expressed across deep-layers (Figure 3F). This widespread expression limits their specificity for unambiguous identification of CSNs.

To overcome this constraint, we mapped the spatial distribution of tdTomato transcripts, revealing a sparse but highly specific signal within layer V (Figure 3G). As expected for sequencing-based detection, transcript capture was incomplete, so we complemented this approach with fluorescent detection of tdTomato protein, which provided a more comprehensive representation of labeled CSNs (Figure 3H). By integrating transcriptomic and protein-level tdTomato information, we manually defined regions of interest and selected the corresponding spots for downstream analysis (Figures 3H, 3I). To independently verify our selection strategy, we trained the another model with our previously generated scRNAseq data of bona fide adult CSNs^10^ and found high-confidence prediction of CSN identity for most spots (Figure 3J). We then examined the heterogeneity within these CSN-enriched spots. Deconvolution restricted to this subset revealed representation of the various CSN subtypes, including CSN-IT, -PT, -CT, and -NP populations (Figures 3K, 3L). Notably, integration of CSN identity with global cortical clustering illustrates the abundance and spatially restricted distribution of CSNs among other cortical cell types (Figure 3L’). Together, combining scRNAseq–based deconvolution with retrograde labeling overcomes the limitations of canonical marker genes and enables precise identification of not only cortical layers, but specifically CSN subpopulations within our spatial transcriptomic data for differential expression analysis.

### Injured and spared CSNs exhibit distinct transcriptional signatures.

**Figure 4.**
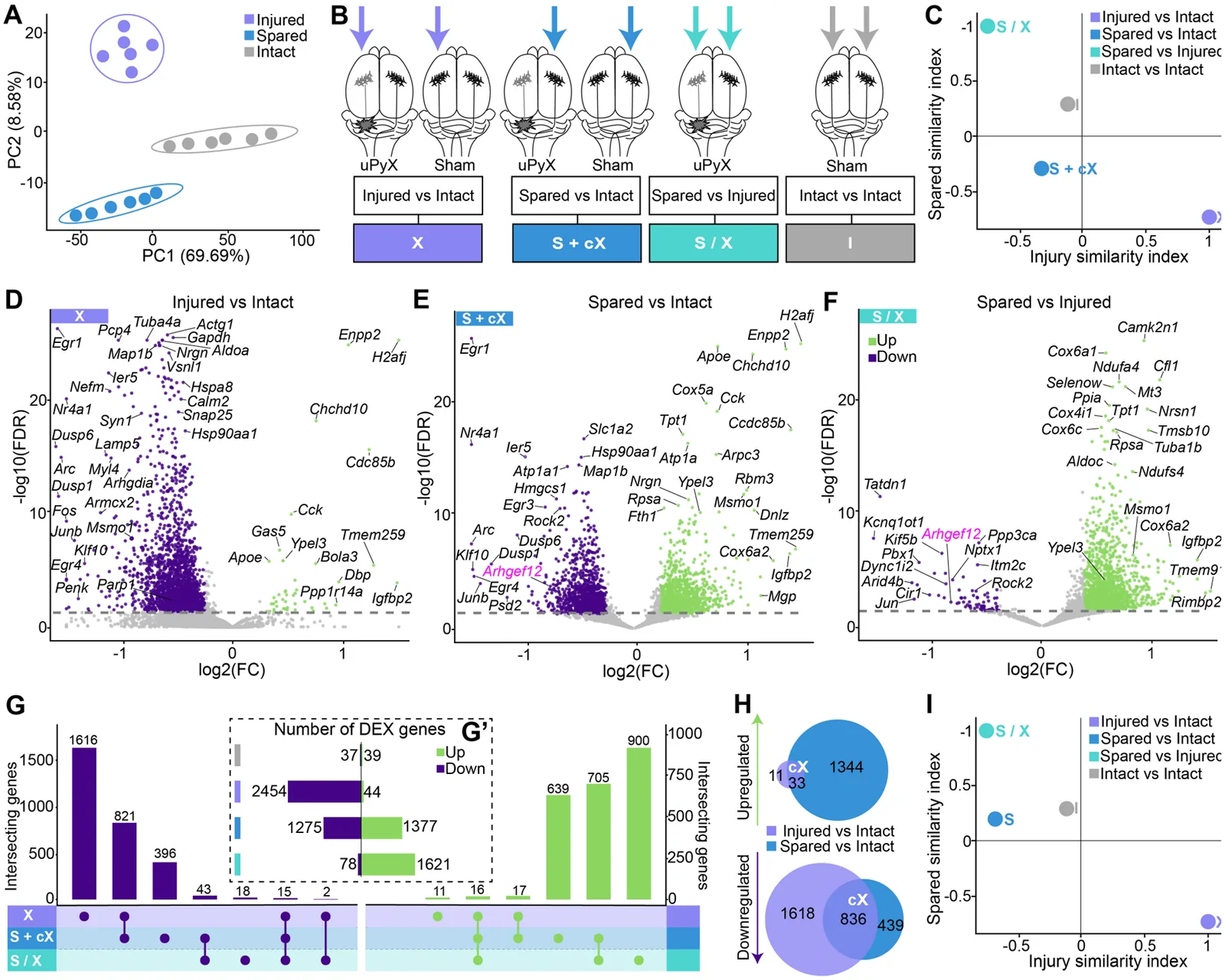
Injured and spared CSNs exhibit distinct transcriptional signatures. **(A)** PCA of CSNs pseudobulk-normalized gene expression from CSN-enriched spots per animal. n = 6 mice per group. **(B)** Schematic of differential expression contrasts performed. **(C)** Similarity-space projection of differential expression contrasts using injury and spared transcriptional axes. **(D–F)** Volcano plots showing differentially expressed genes for injured versus intact **(D)**, spared versus intact **(E)**, and spared versus injured **(F)**. **(G)** Intersection of differentially expressed genes among contrasts. **(G′)** Number of significantly upregulated and downregulated genes in each comparison. **(H)** Overlap of differentially expressed genes between injured and spared CSNs, highlighting injury-context (cX) genes. **(I)** Similarity-space projection following computational removal of shared injury-context genes from the spared versus intact comparison. Differential expression was assessed using the Wald test implemented in PyDESeq2, with Benjamini–Hochberg correction for multiple comparisons. Differentially expressed genes were defined by padj ≤ 0.05 and |log2(FC)| ≥ 0.25.

Next, we examined how injury status impacts the transcriptional states of CSNs by comparing gene expression of intact, injured, and spared CSNs (Figure 4). Principal component analysis (PCA) of normalized counts revealed clear segregation of samples by injury status. PC1 robustly separated injured (uPyX) from intact (sham) animals, while PC2 divided the spared from the injured CSNs within uPyX animals (Figure 4A). Crucially, this separation acts as an internal check for lesion completeness because if a lesion was incomplete, putative injured CSNs would cluster closer to the spared ones, which was not the case for any animal.

We next utilized these distinctions to conduct all biologically meaningful pairwise contrasts between the 3 populations (Figure 4B). First, injured vs intact CSNs (purple, X) were compared to isolate the injury-induced transcriptional program. Second, spared vs intact CSNs (blue, S + cI) were compared to capture spared-related changes within the broader injury context experienced by the animal. Third, to resolve the spared-specific program, we compared spared vs injured CSNs (cyan, S / X) within uPyX animals. Fourth, we performed a final control comparison between hemispheres within intact animals (grey, I) to account for background variability. To quantify relationships among these contrasts, we projected each differential expression result into a two-dimensional similarity space defined by an “injury axis” (X) and a “spared axis” (S / X) (Figure 4C). The control (I) localized near the origin, indicating minimal similarity to either profile. The spared vs intact (S + cX) comparison occupied a distinct quadrant, consistent with a mixed profile that contains both injury context and spared-associated components. Gene-level differential expression analysis revealed a largely downregulated transcriptome in injured CSNs (Figure 4D), while spared CSNs showed both up- and downregulation (Figure 4E).

Notably, there was a substantial overlap of DEGs among the injured vs intact (X) and spared vs intact (S + cX) contrasts (Figures 4D, 4E). These genes encompassed transcriptional regulators, activity-responsive genes, and stress-associated phosphatases, including Klf10, Nr4a1, Egr1, Egr4, Arc, Junb, Dusp1, and Dusp6. The spared vs injured (S / X) contrast reflected a distinct pattern with a predominantly upregulated transcriptome in spared CSNs and minimal overlap with dysregulated genes from the injured vs intact (X) contrast (Figure 4F).

We quantified the amount of overlap between contrasts by counting the number of DEGs (Figure 4G). We found 2,498 DEGs in the injured vs intact (X, down = 2,454; up = 44), 2,652 DEGs in the spared vs intact (S + cX, down = 1,275; up = 1,377), 1,699 DEGs in spared vs injured (S / X, down = 78; up = 1,621), and only 76 DEGs in the control intact vs intact contrast (I, down = 37; up = 39) (Figure 4G’). There were 838 common DEGs between the injured vs intact and spared vs intact comparisons with (X and S + Cx, down = 821; 17 = up), and only 31 DEGs common across all comparisons (down = 15; 16 = up) (Figures 4G). We operationally defined genes dysregulated in the same direction in injured vs intact (X) and spared vs intact (S + cX) CSN-enriched spots as shared injury context genes (cX, down = 836; 33 = up) (Figure 4H). These genes were removed from the spared vs intact (S + Cx) set to isolate the spared-specific signature (S), which was used for all downstream analyses. Reprojection of this filtered profile (S) into the similarity space, resulted in a shift toward the spared quadrant (Figure 4I). These analyses demonstrate that injured and spared CSNs engage distinct but partially overlapping transcriptional programs which can be computationally disentangled to reveal a more precise spared-specific transcriptional signature.

### Functional analysis reveals convergence of spared-specific DEGs on plasticity programs.

**Figure 5.**
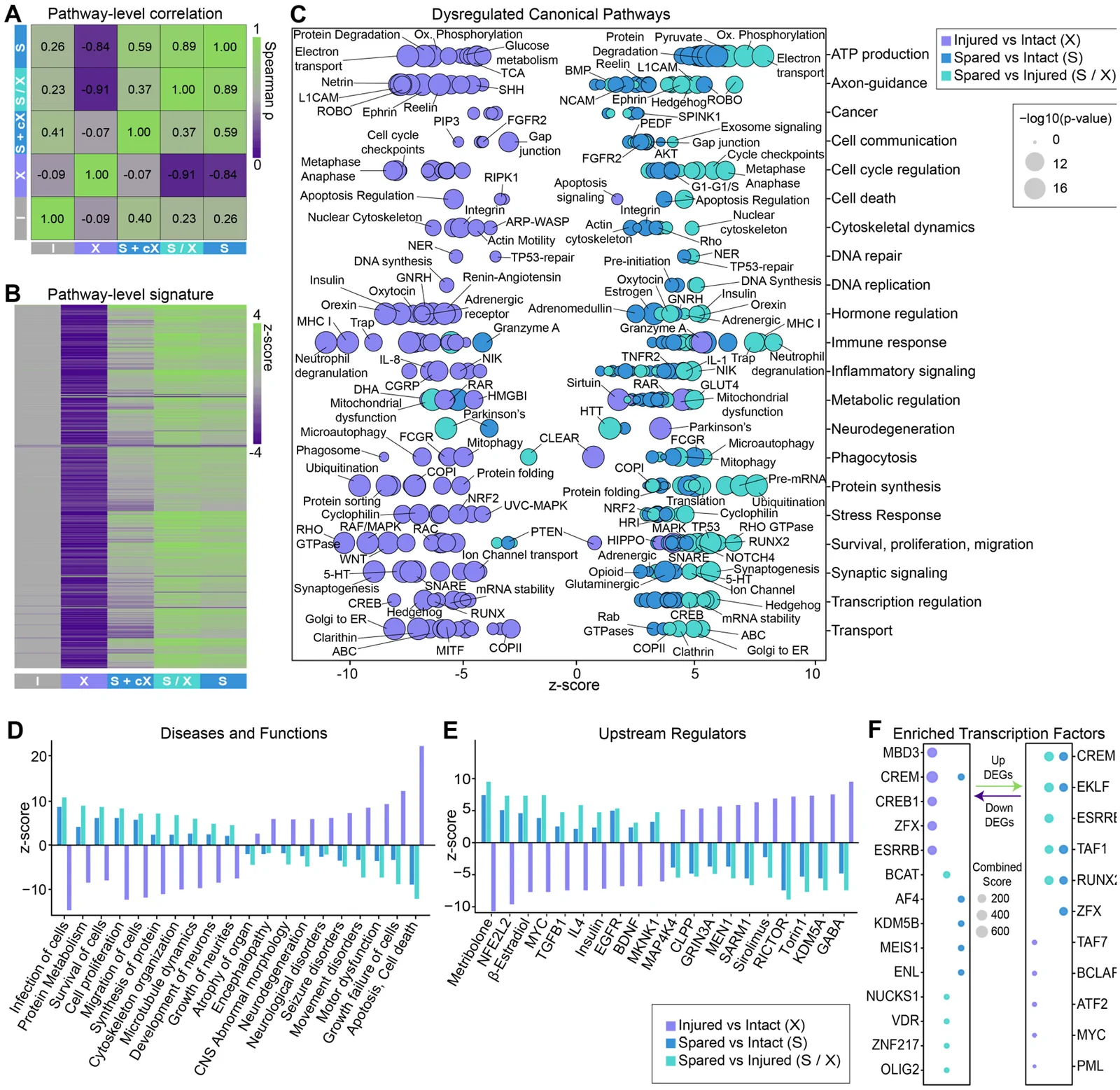
Functional analysis reveals convergence of spared-specific DEGs on plasticity programs. **(A)** Weighted Spearman correlation matrix of canonical pathway activation scores across comparisons. **(B)** Heatmap showing activation z-scores for the 500 most dysregulated *Canonical Pathways*. **(C)** *Canonical Pathways* grouped by broad biological processes. All displayed pathways had a p-value ≤ 0.05. **(D)** Top 10 predicted activated and top 10 predicted inhibited *Diseases and Biological Functions* for each comparison, ranked by activation z-score. All displayed terms had a p-values ≤ 2.29 × 10^−7^. **(E)** Top 10 predicted activated and top 10 predicted inhibited *Upstream Regulators* for each comparison, ranked by activation z-score. All displayed regulators had a p-value ≤ 0.00425. **(F)** Top 5 significantly enriched transcription factors for upregulated and downregulated DEGs in each comparison, ranked by Enrichr Combined Score (padj ≤ 0.05; displayed TF padj range: 4.58 × 10^−100^ - 0.03). DEGs (padj ≤ 0.05, |log2FC| ≥ 0.25) were used for all functional analysis in IPA and Enrichr.

To determine whether gene-level differences between injured and spared CSNs translate into coherent programs, we assessed functional pathway and transcription factor enrichment via Ingenuity Pathway Analysis (IPA)^20^, and Enrichr^21,22^, respectively (Figure 5). At the pathway level the two spared-specific signatures (S and S / X) were highly correlated (*ρ* = 0.89), and both were strongly anticorrelated (*ρ* = −0.84, −0.91) with the injury signature (*X*, Figure 5A). This convergence demonstrates that subtracting injury-context genes effectively isolates the spared-specific program that is directionally opposed to the injury response. Clustering of the top 500 dysregulated IPA *Canonical Pathways* revealed a mixed and attenuated signature in spared vs intact CSNs (*S* + *cX*). The injury profile (*X*) was characterized by strong pathway inhibition, while both spared-specific profiles (*S* and *S / X*) showed widespread activation (Figure 5B). Specifically, *Canonical Pathways* related to metabolic support and biosynthesis (Oxidative Phosphorylation, Electron Transport, and Glucose Metabolism), transcriptional and translational machinery (CREB Signaling, mRNA Stability, Protein Folding), and transport (COPI/II, Clathrin, ABC), were predicted to be activated in spared CSNs. Pathways linked to structural remodeling and connectivity, such as axon guidance (Ephrin, ROBO signaling, Reelin), cell adhesion and communication (NCAM, L1CAM, Gap Junction Signaling), as well as cytoskeletal dynamics (actin cytoskeleton, Rho GTPase signaling), also had high activation scores (Figure 5C). Additionally, Infection of Cells, Protein Metabolism, Cell Survival, Cytoskeletal Organization, Microtubule Dynamics, and Growth of Neurites were the most enriched *Diseases and Biological Functions* in spared CSNs, which further supports a growth-permissive, pro-plasticity state. (Figure 5D). In contrast, injured CSNs exhibited selective predicted activation of stress and cell death *Canonical Pathways* (Mitochondrial Dysfunction, Apoptosis, Parkinson’s Signaling, HIPPO), coupled with Growth Failure, and Motor Dysfunction as the most enriched *Diseases and Biological Functions* (Figures 5C, 5D).

Using IPA *Upstream Regulators* analysis, we found activation of the metabolic regulator MYC, ROS-induced damage regulator NFE2L2, and anti-inflammatory cytokines TGFB1 and IL-4. Growth-promoting hormonal and trophic signaling, including β-estradiol, insulin, androgen receptor signaling (Metribolone), EGFR, and BDNF, was also predicted to be activated. These pathways have established roles in promoting neuronal growth and neurite extension, converging in part on PI3K–AKT–mTOR and Ras–Raf–MEK–ERK signaling^23–27^ (Mannella and Brinton, 2006; Castoria et al., 2015; Gonzalez et al., 2016; Romano and Bucci, 2020). Consistent with increased mTOR signaling, the analysis predicted inhibition of mTOR pathway antagonists, Sirolimus (rapamycin) and Torin1. Moreover, several predicted regulatory changes in spared CSNs were consistent with reduced constraints on neuronal growth and plasticity. These included suppression of the chromatin-associated repressor KDM5A, a chromatin-associated H3K4 demethylase whose degradation promotes neurite outgrowth^28^, the axon degeneration-associated NAD+ hydrolase SARM1^29,30^, and the neurite degeneration-associated kinase MAP4K4^31^, together with modulation of GABAergic inhibitory neurotransmission, a key regulator of cortical plasticity and circuit reorganization^32^.

Additionally, transcription factor enrichment analysis predicted activity-dependent and pro-plasticity regulators, CREM^33^, ESRRB^34^, RUNX2^35^, and EKLF^36^ to be enriched in spared CSNs, while chromatin-associated repressor ZNF217^37^ was predicted to be de-enriched (Figure 5F). In injured CSNs, enriched transcription factors included BCLAF1^38^, PML^39^, and the classic injury response factor ATF2^40^ (Figure 5F). Together, this hierarchical analysis allowed us to link gene-level changes to the higher-order regulatory architecture underlying each transcriptional state.

### Network-based prioritization identifies *Arhgef12* as a negative regulator of neurite regeneration.

**Figure 6.**
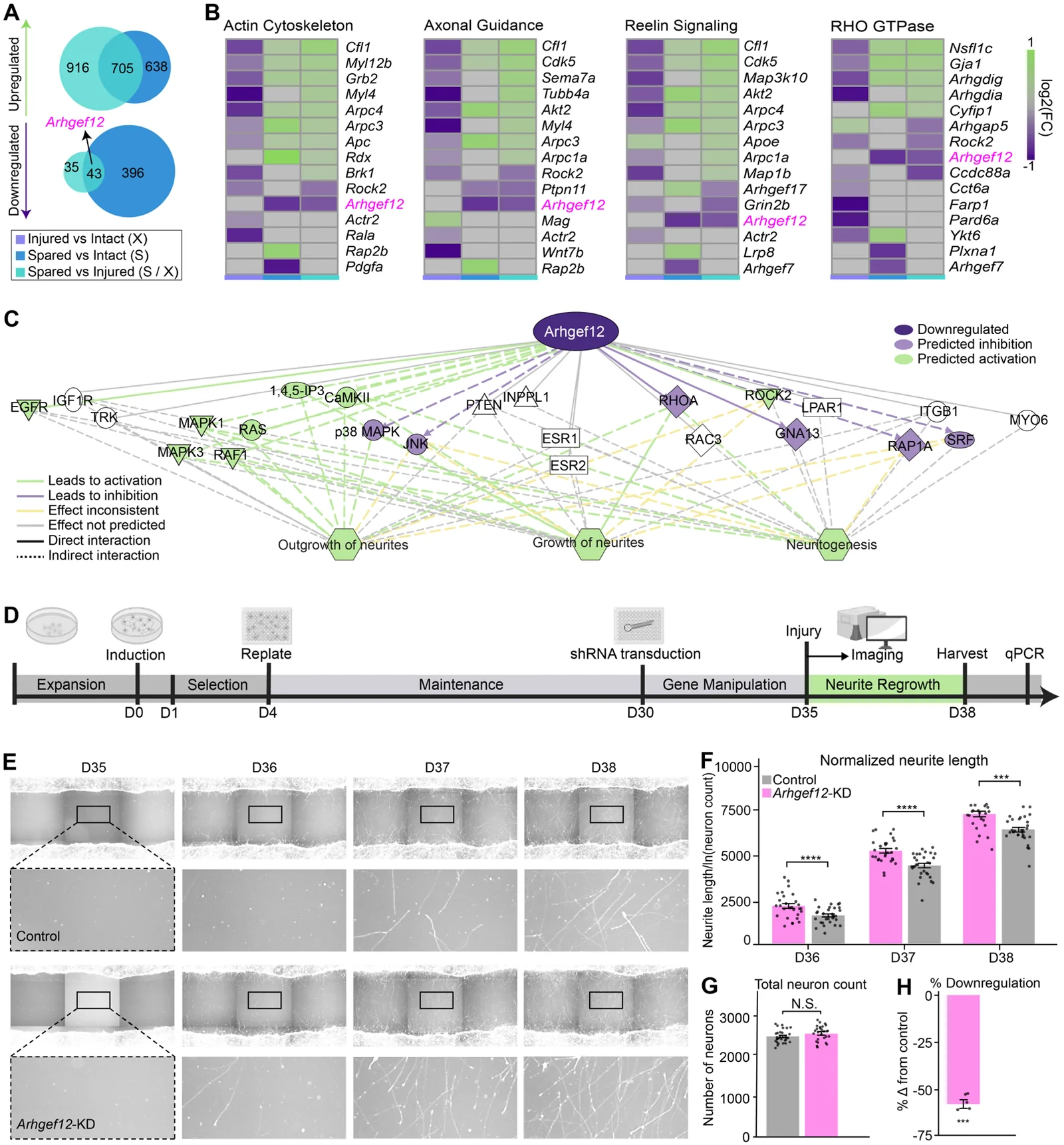
Network-based prioritization identified *Arhgef12* as a negative regulator of neurite regeneration. **(A)** Intersection of differentially expressed genes shared between the two spared-specific comparisons. Significance threshold: padj ≤ 0.05 and log2(FC) ≥ |0.25| **(B)** Heatmaps showing the top 3 upregulated and top 3 downregulated genes from each contrast within significantly dysregulated structural plasticity pathways. Actin Cytoskeleton Dynamics (p-values: S, 9.1 × 10^−8^; S / X, 4.8 × 10^−6^), Axonal Guidance (p-values: S, 3.2 × 10^−10^; S / X, 1.1 × 10^−7^), Reelin signaling (p-values: S, 4.3 × 10^−8^; S / X, 8.5 × 10^−7^), and Rho GTPase signaling (p-values: S, 4.4 × 10^−10^; S / X, 2.9 × 10^−9^). **(C)** IPA MAP network illustrating predicted effects of *ARHGEF12* downregulation on neuritogenesis and growth of neurites. **(D)** Experimental workflow and timeline of neurite regeneration assay. **(E)** Representative images of neurite regeneration following mechanical injury in control (top) and *ARHGEF12*-KD (bottom) neurons. Insets show higher-magnification views of the regenerating region. **(F)** Quantification of normalized neurite length with post hoc comparisons between *ARHGEF12*-KD and control at each day following injury. **(G)** Total neuron counts at the experimental endpoint. **(H)** qPCR quantification of *ARHGEF12* expression as a percent of the control. Data are presented as mean ± SEM. Neurite regeneration: control (n = 30 wells), *ARHGEF12*-KD (n = 28 wells); qPCR: n = 5 per condition. Statistical significance: ns, not significant; *P* < 0.05 (*), *P* < 0.01 (**), *P* < 0.001 (***), *P* < 0.0001 (****).

To identify candidate pro-plasticity regulators, we performed a network-based prioritization that systematically refined our DEG list to genes that were shared between both spared-specific (S and S / X) comparisons and consistently represented in pathways related to axonal growth and repair (Figure 6). We began by taking the intersection of DEGs in the spared-specific comparisons (S and S / X) to establish a high-confidence list that consisted of 705 up- and 43 down-regulated genes (Figure 6A).

We next focused on structural plasticity *Canonical Pathways* that were significantly enriched across both spared-specific comparisons, including Actin Cytoskeleton Dynamics, Axonal Guidance, Reelin Signaling, and Rho GTPase Signaling (Figure 6B). We found increased expression of cytoskeletal and axonal branching genes (*Tubb4a*, Arp2/3 complex subunits) in spared CSNs, whereas components of the RhoA growth-inhibitory signaling cascade (*Rock2*, *Arhgef12*) were consistently among the most downregulated genes. Since *Arhgef12* was recurrently present in all these pathways, we used IPA Molecule Activity Predictor (MAP) to better understand the functional impact of *Arhgef12* modulation. Downregulation of *Arhgef12* was predicted to increase neuritogenesis and growth of neurites (Figure 6C). These effects were consistent with reduced RhoA signaling (*Lpar1*, *Gna13*, *Arhgef12*, *Rhoa*, *Rock2*), a well-established inhibitory axis for axonal growth^41,42^. Beyond RhoA signaling, the MAP network connected Arhgef12 modulation to receptor tyrosine kinase–MEK–ERK signaling (EGFR, IGF1R, Trk, Ras and Raf), calcium-dependent signaling (IP3 and CaMKII), JNK and p38 MAPK signaling, and regulators of PI3K signaling and estrogen-receptor activity (PTEN, INPPL1 and ESR1/2). Additional predicted interactions involved adhesion-associated signaling (RAP1A and ITGB1), SRF-dependent cytoskeletal regulation and MYO6-associated intracellular transport. Together, these in silico predictions nominated Arhgef12 as a putative negative regulator of axonal growth.

To validate whether *ARHGEF12* plays an inhibitory role in axonal growth in a human neuronal model, we conducted a loss-of-function experiment in human induced pluripotent stem cell (iPSC)-derived *NGN2*-induced functionally mature glutamatergic neurons^43,44^ (Figure 6D). At 30 days *in vitro* (DIV) neurons were transduced with lentiviral shRNA constructs for *ARHGEF12* or a non-targeting control. On 35 DIV neurons underwent a mechanical transection, followed by high-content imaging and neurite tracing for 3 days. Representative images demonstrated consistent injury across conditions (Figure 6E).

Quantitative analysis using a linear mixed-effects model revealed a significant increase in normalized neurite length in *ARHGEF12*-KD neurons compared with controls at each day following injury (Figure 6F). Critically, total neuron number did not differ between groups (Figure 6G), indicating that enhanced neurite regeneration was not due to differences in neuronal survival or plating density. We then confirmed that *ARHGEF12* expression was reduced by 59.6% relative to the control via qPCR (Figure 6H). Together, these findings provide functional validation of the computational predictions and identify *ARHGEF12* as a negative regulator of neurite regeneration in human neurons.

### Drug perturbation analysis identifies vorinostat as a pharmacological enhancer of neurite regeneration.

**Figure 7.**
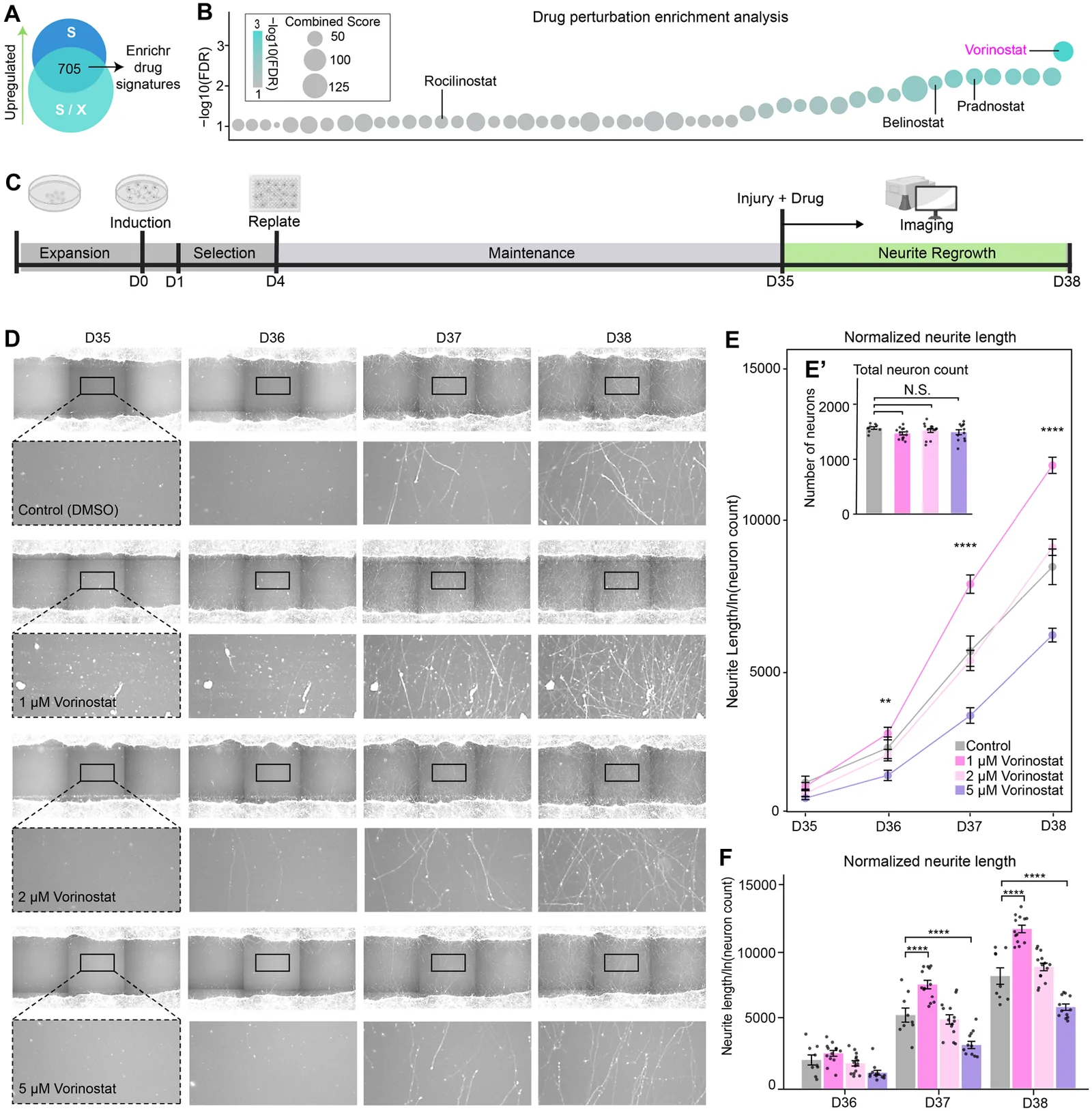
Drug perturbation analysis identifies vorinostat as a pharmacological enhancer of neurite regeneration. **(A)** Drug perturbation enrichment workflow using the 705-gene common spared-specific transcriptional signature as input for the LINCS L1000 Chemical Perturbations up library. **(B)** Top enriched drug perturbation signatures ranked by padj. Bubble size represents the Enrichr combined score. vorinostat and other HDAC inhibitors are highlighted. **(C)** Experimental workflow and timeline of the pharmacological neurite regeneration assay. **(D)** Representative images of neurite regeneration following treatment with vehicle (DMSO), 1 μM, 2 μM, or 5 μM vorinostat. Insets show higher-magnification views of the regenerating region. **(E)** Linear mixed-effects model showing normalized neurite length following injury. **(E′)** Total neuron counts at the experimental endpoint. **(F)** Post hoc comparisons between each vorinostat concentration and vehicle control for normalized neurite length at each day following injury. Data are presented as mean ± SEM. Neurite regeneration: Control (n = 9 wells), 1 μM vorinostat (n = 13 wells), 2 μM vorinostat (n = 14 wells), 5 μM vorinostat (n = 12 wells). Statistical significance: ns, not significant; P < 0.05 (*), P < 0.01 (**), P < 0.001 (***), P < 0.0001 (****).

To identify pharmacological strategies predicted to engage components of the endogenous pro-plasticity program observed in spared CSNs, we performed drug perturbation enrichment analysis using the common spared-specific transcriptional signature (S and S / X, Figure 7A). The 705 upregulated genes were queried in Enrichr against the *LINCS L1000 Chemical Perturbations Up* library^21,22,45^ to identify compounds whose induced gene expression profiles significantly overlapped the endogenous plasticity program (Figure 7B). Histone deacetylase (HDAC) inhibitors were prominently enriched among the highest-ranking compounds, with vorinostat emerging as the top candidate based on significance and combined score.

To determine whether vorinostat promotes neurite regeneration, we performed a pharmacological perturbation experiment in human iPSC-derived cortical neurons (^43^, Figure 7C). Mechanical transection was performed on 35 DIV, immediately followed by treatment with vehicle (DMSO) or vorinostat at 1, 2, or 5 μM. Qualitative assessment showed progressively greater neurite regeneration following treatment with 1 μM vorinostat compared with vehicle-treated neurons, whereas higher concentrations diminished regenerative responses (Figure 7D). Using a linear mixed-effects model we found a significant effect of condition on normalized neurite length for all time points (Figure 7E). Total neuron number was consistent between conditions (Figure 7E′). Post hoc comparisons confirmed increased normalized neurite length in neurons treated with 1 μM vorinostat at 2 and 3 days post-injury. In contrast, 2 μM was indistinguishable from the control, whereas 5 μM significantly reduced neurite regeneration (Figure 7F). Together, these findings demonstrate that transcriptome-guided drug prioritization successfully identified a pharmacological intervention capable of enhancing neurite regeneration in human neurons.

## Discussion

In this study we used a combinatorial strategy including retrograde labeling, spatial transcriptomics, and unilateral pyramidotomy (uPyX) to profile injured and spared corticospinal neurons in primary motor cortex (M1) sections (Figure 1). Robust capture of transcripts, conserved tissue morphology, and integration with scRNAseq datasets enabled identification of cortical layers and CSN-enriched spots from known subpopulations^10,11,19^, including IT, PT, CT, and NP CSNs (Figures 2, 3). Differential expression and pathway analysis of CSN-enriched spots revealed distinct and opposing transcriptional states in injured and spared populations (Figures 4, 5). Injured neurons exhibited a largely downregulated profile, enriched for cell death, motor dysfunction, and metabolic imbalance. In contrast, spared neurons exhibited a predominantly upregulated profile characterized by activation of immune, survival, energy production, translation, protein synthesis, and cytoskeletal remodeling programs. Network-based prioritization of genes dysregulated in spared neurons identified *ARHGEF12* as a candidate negative regulator of neurite regeneration (Figure 6), whereas transcriptome-guided drug perturbation analysis identified vorinostat as compound predicted to engage components of the pro-plasticity signature (Figure 7). Consistent with these predictions, *ARHGEF12* suppression and low-dose (1 μM) vorinostat treatment independently enhanced neurite regeneration in human induced pluripotent stem cell (iPSC)-derived cortical neurons.

### Distinct intrinsic states shape the transcriptional responses of injured and spared CSNs to common injury cues.

The main distinction between injured and spared CSNs is the loss of the primary descending spinal axon in the injured population. Axotomy removes the neuron's largest cellular compartment, eliminating the extensive corticospinal projection and the continuous biosynthetic and energetic investment required for its maintenance. Consistent with this structural change, injured CSNs exhibited widespread inhibition of ATP production, metabolic regulation, protein synthesis and transport pathways (Figure 5C). Additionally, injured CSNs remain embedded within cortical and thalamocortical networks that could convey activity-dependent or trophic signals after distal axotomy^46,47^. However, the loss of their principal motor output creates an imbalance between preserved synaptic input and functional output. Together, these structural, metabolic, and activity-dependent changes may fundamentally reshape the intrinsic state of injured CSNs.

In contrast, spared CSNs retain intact connectivity, preserving both synaptic input and functional output. Despite being anatomically uninjured, they are exposed to altered neuronal activity arising from injury-induced network reorganization, including interhemispheric signaling^48,49^, as well as extrinsic cues associated with the injury response^50^. Following injury, activation of resident glia, infiltration of peripheral immune cells, and the release of cytokines and chemokines initiate a signaling cascade that extends throughout the CNS^51^. In addition to promoting debris clearance and wound healing, this immune response can induce transcriptional reprogramming in both local and distal cells^52,53^. Consistent with this concept, immune and anti-inflammatory pathways were among the most enriched biological programs in spared CSNs (Figures 5C, 5E). Yet, unlike injured CSNs, the preserved structural integrity and potentially increased network activity in spared CSNs likely underlies their coordinated upregulation of metabolic, survival, translational, and cytoskeletal remodeling pathways in response to injury-associated signals (Figures 5C, 5E). Together, these findings suggest that depending on their intrinsic states, common extrinsic cues may contribute to degeneration in injured neurons while simultaneously priming spared CSNs to activate adaptive plasticity programs.

### Spared CSNs integrate metabolic, structural, and survival programs to support plasticity.

Spared CSNs exhibited a highly permissive transcriptional state characterized by coordinated activation of biological programs that support not only neuronal survival but also structural and functional reorganization after injury. If spared CSNs receive increased excitatory drive from contralateral cortical circuits following unilateral injury, the initial increase in synaptic output would likely rely on pre-existing cellular machinery. Sustained structural remodeling, however, requires increased energy production together with enhanced biosynthetic capacity. Consistent with this concept, GABA Signaling was among the most de-enriched *Upstream Regulators* in spared CSNs (Figure 5E), whereas the activity-dependent transcription factor CREM and the metabolic regulators MYC and ESRRB were among the most enriched (Figure 5E, 5F). These changes were accompanied by coordinated activation of pathways involved in metabolic regulation, transcription, protein synthesis, and intracellular trafficking (Figure 5C, 5D). Spared CSNs also showed enrichment of transcription factors (EKLF, RUNX2) and pathways involved in axon guidance, cell communication, cytoskeletal dynamics, and neurite growth (Figure 5C, 5D), supplying the structural components for collateral sprouting. Simultaneously, activation of anti-inflammatory, antioxidant, and pro-survival regulators, including NFE2L2, TGFB1, IL4, mTOR, and BDNF (Figure 5C–E), is consistent with the maintenance of cellular homeostasis and preservation of neuronal integrity. Similar metabolic and antioxidant programs have previously been identified in regenerating CSNs following *Pten/Socs3* deletion^5^, further supporting the importance of mitochondrial function and cellular metabolism during axon repair. Rather than representing independent biological processes, these transcriptional programs appear to function as complementary components of a unified adaptive response, aimed at protecting and strengthening existing circuits, while building new connections after injury.

### Endogenous plasticity programs are detectable even if insufficient to drive functional recovery.

Despite the robust pathway-level pro-plasticity signature observed in spared CSNs, spontaneous anatomical and functional recovery after injury is limited^54,55^. Several factors may contribute to the disconnect between the observed transcriptional response and the biological outcome. First, the magnitude of individual gene expression changes remained relatively modest, with most differentially expressed genes in both spared and injured CSNs exhibiting log2(FC) < 1. Although our previous study primarily investigated sprouting CSNs in *Ngr1*-null mice, the accompanying wild-type comparison between spared and injured CSNs after uPyX also identified minimal dysregulation^4^. Similarly, a separate study of injured CSNs reported limited transcriptional changes one week after cervical and thoracic spinal cord injury^7^. Our injured CSNs recapitulated the downregulation of activity-dependent genes including Egr1, Egr4, Arc, Nr4a1, and Junb (Figure 4D), together with enrichment of injury-responsive transcription factors such as ATF2 (Figure 5F). These concordant findings support the biological identity and relevance of the molecular signatures of injured and spared CSNs, while reinforcing the concept that the magnitude of these endogenous responses to distal axotomy may be insufficient to drive substantial functional recovery.

Second, the endogenous pro-plasticity state may be inherently transient. Consistent with this idea, previous characterization of layer V neurons showed that injury alone can induce an early regenerative transcriptome characterized by reversion toward an embryonic-like state^6^. Our spared CSNs exhibited transcriptional features consistent with an immature, growth-permissive state, including increased HTT upregulation (Figures 5C, 5D). However, in that study in the absence of neural progenitor cell grafts, this transcriptional program progressively declined after day 10. Likewise, we previously demonstrated that untreated mice undergo minimal anatomical reorganization and spontaneous recovery beyond modest initial improvements during the first week post-injury, while motor rehabilitation significantly enhanced corticospinal remodeling and continued functional recovery^56^. Collectively, these findings suggest that intrinsic plasticity programs may require external reinforcement to be sustained.

Third, our analysis suggests that the endogenous pro-plasticity response of spared CSNs is constrained by concurrent injury-associated signaling (Figures 4E, 4H). Although computational removal of shared injury-context genes (*S - cI*) revealed a robust pro-plasticity transcriptional signature, both programs coexist within spared neurons *in vivo*. Consequently, injury-associated transcriptional responses can counterbalance activation of plasticity-related pathways, producing a more muted intrinsic growth state (Figure 5B). Importantly, these intrinsic constraints are compounded by the inhibitory extracellular environment that develops after CNS injury. Reactive gliosis and scar formation generate a complex lesion environment in which chondroitin sulfate proteoglycans, myelin-associated inhibitors, and persistent neuroinflammation can restrict axonal growth and structural remodeling^57–59^. Thus, even when spared corticospinal neurons initiate an endogenous growth program, this response must compete with injury-associated intrinsic signaling and multiple extrinsic inhibitors. Together, these observations support a model in which spontaneous corticospinal plasticity is constrained at multiple levels: magnitude, duration, and concurrent intrinsic and extrinsic injury-inhibitory signals.

### An integrative framework for target- and state-based therapeutic discovery.

Our integrative discovery pipeline demonstrates how transcriptional profiling of spared CSNs can be leveraged to identify both intrinsic genetic regulators and pharmacological interventions that promote neurite regeneration. Our gene prioritization strategy included refinement at multiple levels: isolation of the spared-specific signature, overlapping gene lists from between and within animal comparisons, recurrence of candidates at the pathway-level, and *in silico* predictions of their involvement in neurite growth. Through this approach, *Arhgef12* emerged as a central negative regulator of neurite regeneration. Although *Arhgef12* has been primarily characterized as a Rho guanine nucleotide exchange factor that activates RHOA^60–62^, our analyses predicted broader interactions with multiple cytoskeletal remodeling pathways. Consistent with these predictions, *ARHGEF12* suppression significantly enhanced neurite regeneration in human iPSC-derived cortical neurons, establishing *ARHGEF12* as a previously unrecognized regulator of axonal regeneration.

In parallel, unbiased drug perturbation analysis^21,22,45^ identified histone deacetylase inhibitors as a convergent pharmacological class associated with the spared-CSN transcriptional program. Vorinostat was the highest-ranked compound, while the independent enrichment of pracinostat, belinostat and rocinostat demonstrated convergence on HDAC inhibition as a pharmacological class rather than an isolated compound-signature match. HDAC inhibitors have previously shown beneficial effects in experimental stroke and spinal cord injury, where their actions have been linked predominantly to neuroprotection, modulation of inflammatory and oxidative signaling^63–65^, but also to enhanced neurite outgrowth and axon regeneration^66,67^. Vorinostat itself has demonstrated neuroprotective effects following cerebral ischemia and hemorrhage^63,68^, promotes neurite outgrowth in neuronal models^69^, and has been evaluated following traumatic spinal cord injury^70^. Here, rather than selecting HDAC inhibition *a priori*, our unbiased analysis of spared CSNs independently nominated vorinostat as a compound predicted to engage components of the endogenous pro-plasticity program, while subsequent validation in human neurons demonstrated a direct effect on neuronal growth competence. These results illustrate how complementary target- and state-based strategies can be identified and validated in a neuronal model that provides initial evidence of their efficacy in a human genetic background. These results illustrate how complementary target- and state-based strategies can be identified and validated in a neuronal model that provides initial evidence of their efficacy in a human genetic background.

### Limitations of the study

#### Injury Paradigm

The uPyX model permits selective unilateral targeting of the corticospinal tract at a distal location while preserving the contralateral side and adjacent cortical circuitry^4,71^. Although a uPyX does not fully recapitulate clinically relevant spinal cord injury, it was well suited for transcriptional profiling of a specific neuronal population within a single animal across two distinct states: injured and spared.

#### Spatial Resolution

Spatial transcriptomics enables capture of nuclear and cytoplasmic transcripts directly from the tissue circumventing the need for dissociation and avoiding processing-related gene expression artifacts. This approach combined with retrograde labeling from the spinal cord and reference scRNAseq atlases for deconvolution affords cell type specificity mitigating the limitation of the non-single cell resolution. Moreover, preservation of anatomical architecture can turn resolution constraints into an advantage by including information not only from the target CSNs but also from their adjacent native microenvironment, which can better reflect complex biological states, such as intrinsic gene expression changes of spared neurons coexisting with injury-induced contextual cues. Future studies, at single-cell spatial resolution, thanks to newly released technologies (Visium HD), will allow explicit interrogation of gene expression changes driven by each cell type, including astrocytes and microglia.

#### Temporal Resolution

This study focuses on a single time point (D10) after injury. We selected D10 because endogenous gene expression changes after spinal cord injury have been reported to persist through this stage^6,7^. In addition, we previously observed the onset of spontaneous functional recovery between D7 and D14 after injury^56^, suggesting that transcriptional programs driving circuit reorganization are likely initiated before or during this period. Consistent with this timeline, we also found that motor rehabilitation promotes anatomical reorganization and significantly enhances functional recovery^56^. Future studies will extend these findings by profiling rehabilitation-induced gene expression across multiple post-injury time points to better define the temporal sequence of molecular events underlying spontaneous and rehabilitation-induced plasticity.

In summary, we found that spared CSNs mount an endogenous pro-plasticity response after unilateral injury. This transcriptional signature can be leveraged to identify target and state-based repair strategies such as *ARHGEF12* and vorinostat. Whereas ARHEGF12 suppression engages a defined target linked to RHOA and cytoskeletal remodeling, vorinostat acts at the state level across transcriptional programs associated with the pro-plasticity response. Their independent effects in human cortical neurons support the biological relevance of the spared-CSN transcriptional signature. More broadly, these findings establish a scalable framework integrating spatial transcriptomic discovery with functional screening in human neurons to translate endogenous responses to CNS injury into candidate strategies for neural repair.

## Methods

### Animals

Adult C57BL/6J mice (The Jackson Laboratory) (n = 12; 10 females and 2 males) were randomly assigned to receive unilateral pyramidotomy (uPyX; n = 6) or sham surgery (n = 6). Animals were housed under standard laboratory conditions with *ad libitum* access to food and water and maintained on a 12 h light/dark cycle. All procedures were approved by the Yale University Institutional Animal Care and Use Committee (IACUC) and performed in accordance with NIH guidelines.

### Human Induced Pluripotent Stem Cell (iPSC) Lines

A previously established and characterized human induced pluripotent stem cell (hiPSC) line, NSB2607 clone 2 (2607–2; karyotype XY), was used in this study^72^. Previously generated cryopreserved Stem cell-derived NGN2-accelerated neural progenitor cells (SNaPs)^73^ derived from the hiPSC line above, were obtained from the Brennand laboratory and used for all experiments. Yale University determined that experiments using these de-identified human iPSC-derived cells did not constitute human subjects research and therefore did not require Institutional Review Board approval. Cell cultures were routinely tested and confirmed negative for mycoplasma contamination.

### Retrograde labeling

Adult C57BL/6J mice underwent bilateral retrograde labeling of corticospinal neurons (CSNs) by intraspinal injection of AAVrg-CAG-tdTomato. Mice were anesthetized by intraperitoneal injection of ketamine (100 mg/kg) and xylazine (10 mg/kg). The adequacy of anesthesia was confirmed by the absence of corneal and toe-pinch reflexes. Ophthalmic lubricant was applied to both eyes to prevent corneal drying during surgery. Mice were secured in a stereotaxic frame using atraumatic ear bars, and the surgical site was shaved, scrubbed with betadine, and swabbed with 70% ethanol. A bilateral laminectomy was performed at either the cervical (C6/C7) or lumbar (L4/L5) spinal cord. A pulled glass capillary attached to a 5 μL Hamilton syringe mounted on a Micro4 infusion pump (World Precision Instruments) was positioned using stereotaxic guidance and inserted to a depth of 500 μm and 600 μm lateral to the midline. 30s after insertion, 100 nL of AAVrg-CAG-tdTomato was infused over 2 min at each injection site. The capillary was left in place for an additional 30s before being slowly withdrawn to minimize reflux. This procedure was repeated 7x at evenly spaced injection sites. The surgical site was closed with Vicryl absorbable sutures, and animals received postoperative analgesia (buprenorphine, 0.05 mg/kg, subcutaneous) and antibiotics (ampicillin, 100 mg/kg, subcutaneous) for 2 days after surgery. Mice recovered for 14 days to allow retrograde transport to the cortex and robust tdTomato expression prior to uPyX or sham surgery.

### Unilateral pyramidotomy (uPyX)

Mice were anesthetized as above. Mice were positioned in the supine position, ophthalmic lubricant applied to both eyes and the neck area shaved and scrubbed with betadine and alcohol. A ventral midline incision was made to the left of the trachea. Blunt dissection was performed to expose the occipital bone at the base of the skull. The occipital bone was carefully removed on the left side of the basilar artery using blunt Dumont #2 forceps to expose the medullary pyramids. The dura mater was pierced with a 30-gauge needle and resected. The left medullary pyramid was transected unilaterally to a depth of 0.25 mm using fine Dumont #5 forceps, whereas sham-operated animals underwent identical surgical exposure without transection of the pyramid. No internal sutures were placed, and the skin incision was closed using monofilament sutures. Animals received postoperative analgesia (Buprenorphine, 0.05mg/kg subcutaneously) and antibiotics (Ampicillin, 100mg/kg subcutaneously) for 2 days post-surgery and were monitored daily throughout recovery.

### Tissue preparation for Visium spatial transcriptomics

Ten days following unilateral pyramidotomy (uPyX) or sham surgery, mice were deeply anesthetized with isoflurane until loss of the toe-pinch reflex and transcardially perfused with ice-cold 0.9% saline supplemented with heparin. Brains were rapidly removed and immediately transferred to ice-cold saline for dissection. Using sagittal and coronal brain matrices, the forebrain was trimmed by making a coronal cut 3 mm caudal to the olfactory bulb followed by sagittal cuts 3 mm lateral to the midline on each hemisphere, thereby isolating a tissue block containing the primary motor cortex (M1). Tissue blocks were embedded in Tissue-Plus O.C.T. Compound, snap frozen on powdered dry ice, and stored at −80°C until cryosectioning. Prior to sectioning, tissue blocks were equilibrated in a cryostat maintained at −12°C for 30 min. Blocks were trimmed to the level of M1 using tdTomato fluorescence as a guide and scored with a razor blade below the corpus callosum to enable placement of two sections within a single 6.5 × 6.5 mm Visium capture area. The two M1 coronal sections (10 μm) from each animal were collected onto a room temperature Visium Spatial Gene Expression v1 Slide^8,74^. Sections from uPyX and sham mice were distributed equally on 3 Visium slides (Slides A-C) to control technical variability, with two sections included per animal within each capture area. Immediately after collection, sections were fixed in pre-chilled 100% methanol for 30 min at −20°C.

### Immunofluorescence

Immediately following fixation, tissue sections were processed for immunofluorescence according to the ‘Methanol Fixation, Immunofluorescence Staining & Imaging for Visium Spatial Protocols’ (CG000312). All solutions, including blocking buffer, primary and secondary antibody solutions, wash buffer, SSC, and mounting medium, were prepared according to the manufacturer's instructions. To preserve RNA integrity, the recommended blocking and antibody incubation times were reduced. The primary antibody solution contained rabbit anti-mCherry antibody (1:1000), and the secondary antibody solution contained mouse anti-NeuN monoclonal antibody (3A4C1), CoraLite Plus 488 (1:100), goat anti-rabbit IgG Alexa Fluor 546 (1:1500), and DAPI (1:3000). All incubations were performed at room temperature. Briefly, sections were incubated in blocking buffer for 5 min, followed by incubation in primary antibody solution for 10 min. After 5x washes, sections were incubated in secondary antibody solution for 10 min, washed an additional 5x, immersed 20X in SSC solution, and cover slipped.

### Microscopy

Whole-slide fluorescence images were acquired immediately following immunostaining using a the Leica upright epifluorescence microscope DM5500 equipped with a 10X objective, following the Visium imaging workflow (CG000241). Identical acquisition settings were used for all slides to ensure consistent fluorescence intensity across samples. Following imaging, slides were stored overnight at 4°C prior to tissue permeabilization and library preparation.

### RNA quality assessment

Adjacent M1 sections were collected during cryosectioning into 1.5-mL microcentrifuge tubes for RNA quality assessment. Total RNA was isolated using the RNeasy Mini Kit (Qiagen) according to the manufacturer's instructions. RNA concentration and purity were initially assessed using a NanoDrop spectrophotometer, and RNA integrity was subsequently determined by the Yale Center for Genome Analysis (YCGA) RNA Quality Control Service using an Agilent 2200 TapeStation to obtain RNA Integrity Number (RIN) values.

### Permeabilization and barcoded cDNA generation

All tissue permeabilization and barcoded cDNA generation were performed according to the Visium Spatial Gene Expression User Guide (CG000239). The optimal tissue permeabilization time for mouse M1 was determined prior to processing experimental samples using the Visium Spatial Tissue Optimization Kit (CG000238). Based on these results, experimental tissue sections were permeabilized for 3 min. Total polyadenylated mRNA was captured by the oligonucleotides within each Visium capture area containing a Poly(Dt) region, spatial barcodes, unique molecular identifiers (UMIs), and TruSeq Read 1 sequencing adapters. Reverse transcription was performed in situ to generate spatially barcoded first-strand cDNA, followed by second-strand cDNA synthesis, denaturation, retrieval of the barcoded free cDNA strand and amplification prior to submission to the YCGA.

### Library preparation and sequencing

Sequencing libraries were prepared by the YCGA according to the Visium Spatial Gene Expression User Guide (CG000239). Library construction included cDNA cleanup, quality control, fragmentation, end repair, A-tailing, double-sided SPRIselect size selection, adaptor ligation, post-ligation cleanup, sample index PCR, post-PCR double-sided size selection, and final library quality control prior to sequencing. Libraries were sequenced on an Illumina NovaSeq platform using paired-end 150-bp sequencing, targeting 177.5 million reads per Visium capture area.

### Spatial sequencing data preprocessing

Raw sequencing data were processed using Space Ranger v1.3.1 (10x Genomics), which aligned fluorescence tissue images and fiducial marker frames to the Visium capture areas and sequencing reads to a custom mm10_tdTomato reference transcriptome to generate spot-by-gene count matrices for each capture area. Count matrices were imported into the scverse Python environment (Python v3.12.9, Scanpy v1.11.5, custom fork of Squidpy based on v1.6.2) for downstream preprocessing and quality control. For each spot, the total UMI counts, number of detected genes, and percentages of mitochondrial and hemoglobin transcripts were calculated. Spots were retained for downstream analyses if they contained <22.5% mitochondrial transcripts, <2.5% hemoglobin transcripts, >1,000 detected genes, and between 1,000 and 30,000 UMIs. Principal component analysis (PCA) was performed on the filtered dataset. To minimize technical variation between Visium slides, batch correction was performed using Harmonypy (v0.0.10), using slide identity as the batch variable. Batch-corrected principal components were used to construct a nearest-neighbor graph, which was subsequently used for UMAP visualization and Leiden clustering. To isolate cortical tissue for downstream analyses, Leiden clusters from each tissue section were projected onto their corresponding spatial coordinates. Based on their spatial localization, clusters corresponding to the cerebral cortex overlying the corpus callosum were identified and recorded for each tissue section. Only spots within the cortical region of interest were retained for subsequent analyses.

### Cell type deconvolution

Cell-type composition of cortical Visium spots was estimated using Stereoscope (scvi-tools) (v1.4.1) with a previously published Allen Institute primary motor cortex (MOp) single-cell RNA sequencing reference dataset^18^. Prior to model training, reference cell populations were balanced by randomly subsampling a maximum of 500 cells per subclass, and subclasses represented by a single cell were excluded. Marker genes were identified within the reference dataset and intersected with genes detected in the Visium dataset. The RNA model was trained for 300 epochs, followed by training of the spatial model for 3,000 epochs. Estimated cell-type proportions were calculated for every cortical Visium spot and used to annotate the major cellular composition of the cortical region. CSN-enriched spots were identified by integrating anatomical location, spatial tdTomato transcript expression, and tdTomato immunofluorescence. Candidate spots located within layer V and associated with retrogradely labeled CSNs were subsequently evaluated using a previously generated adult CSN single-cell RNA sequencing reference dataset^10^ to confirm enrichment for the CSN transcriptional signature. Following validation, the selected spots were subjected to a second round of Stereoscope deconvolution using the Allen Institute primary motor cortex reference dataset^18^ to classify CSN subtypes.

### Assessment of lesion completeness

To assess lesion completeness PyDESeq2 (v0.5.3) normalized gene expression matrices from CSN-enriched spots were exported and pseudobulked by animal. Prior to principal component analysis (PCA), a linear model was used to regress out slide-specific technical effects, including slide × condition interactions, while preserving the biological condition effect. PCA was performed on the 2,000 most variable genes after centering and scaling the corrected expression matrix. The PCA plot was examined to determine whether samples clustered according to experimental condition and to assess inter-animal variability.

### Differential expression analysis

Differential expression analysis was performed using PyDESeq2 (v0.5.3) on raw gene count matrices extracted from CSN-enriched spots. Pairwise comparisons between intact left, intact right, spared, and injured CSN-enriched spots were performed for each Visium slide (Figure 4B). Raw counts were normalized by estimating sample-specific size factors using the DESeq2 median-of-ratios method. Gene-wise dispersions were estimated and negative binomial generalized linear models were fitted for each gene. Differential expression was evaluated using Wald tests. Cook's distance was calculated to identify influential observations, and count outliers were refit when appropriate. Independent filtering and Benjamini-Hochberg correction were applied to adjust for multiple testing. Differential expression results were subsequently post-processed in R (v4.3.3) prior to downstream analyses. Genes associated with technical artifacts, including mitochondrial, ribosomal, hemoglobin, sex chromosome and predicted locus genes, were removed from each dataset. Results from biological replicates were consolidated into a consensus differential expression table by resolving duplicate gene entries according to the largest |log2(FC)|. Genes with padj ≤ 0.05 and |log2(FC)| ≥ 0.25 were considered significantly differentially expressed for downstream analyses and visualization.

### Computational isolation of a spared-specific transcriptional signature

Consensus differential expression signatures were analyzed in R (v4.3.3). Prior to downstream analyses, genes with a baseMean <2 or not detected across all biological comparisons were excluded, and the resulting data were standardized using gene-wise Z-score normalization. The injured (I) and spared (S/I) differential expression signatures (Figure 4B) were used as anchors to define injury and spared similarity indices. Cosine similarity scores between each differential expression signature and the injured and spared anchor signatures were calculated and used to project all biological comparisons onto a two-dimensional injury-spared similarity map. Genes significantly dysregulated in the same direction in both the injured versus intact (I) and spared versus intact (S + cI) comparisons were identified and removed from the spared versus intact comparison to generate a filtered spared-specific signature (S − cI). The filtered signature was subsequently reprojected onto the injury-spared similarity map.

### Functional enrichment analysis

Differentially expressed genes from each comparison were analyzed using Ingenuity Pathway Analysis (IPA, Qiagen; Content Version 165342674) to identify enriched canonical pathways, diseases and biological functions, and upstream regulators. Enrichment significance and predicted activation state were determined using IPA enrichment statistics and activation z-scores. Canonical pathways were grouped into major biological categories for visualization. To compare functional signatures across biological comparisons, weighted Spearman correlations were calculated from IPA pathway activation z-scores, with pathway weights proportional to enrichment significance (−log10(p-value), maximum weight = 50). The resulting correlation coefficients were visualized as similarity matrices. Canonical pathways were subsequently visualized as heatmaps and bubble plots, whereas diseases and biological functions and upstream regulators were ranked according to IPA activation z-scores and displayed as bar plots.

### Transcription factor enrichment analysis

Transcription factor enrichment analysis was performed using Enrichr with the *ChEA 2022* and *ENCODE and ChEA Consensus TFs from ChIP-X* libraries. These libraries identify transcription factors whose experimentally defined target genes are significantly overrepresented among the differentially expressed genes. Upregulated and downregulated genes were analyzed independently for each differential expression signature. Significantly enriched transcription factor terms (padj ≤ 0.05) from both libraries were consolidated by transcription factor, and the highest-ranking transcription factors were used for downstream visualization and comparison across differential expression signatures.

### Candidate gene prioritization

Candidate genes were prioritized by identifying genes significantly dysregulated in the same direction in both spared-specific signatures (S − cI and S/I). Structural plasticity pathways that were significantly enriched in both spared-specific comparisons were then examined, and the top 3 upregulated and top 3 downregulated genes for each contrast ranked by |log_2_FC| were selected from each pathway. Genes recurrently represented across multiple pathways were further evaluated using the Ingenuity Pathway Analysis Molecule Activity Predictor (IPA MAP, Qiagen; Content Version 165342674) to predict the downstream functional consequences of modulating their expression.

### Drug perturbation enrichment analysis

Drug perturbation enrichment analysis was performed using Enrichr with the *LINCS L1000 Chemical Perturbations Up* library to identify compounds whose transcriptional response signatures significantly overlapped the spared neuron-specific transcriptional program. The 705 genes upregulated in the spared-specific transcriptional signature were used as input for the analysis. Enrichment significance was assessed using Enrichr combined scores and Benjamini–Hochberg-adjusted P values. Candidate compounds were ranked according to enrichment score, and the highest-ranked drug was selected for experimental validation.

### Neurite regeneration assay in human iPSC-derived cortical neurons

Human induced pluripotent stem cell (iPSC)-derived cortical neurons were generated from previously established neural progenitor cells^73,75^ by NGN2 overexpression according to previously published protocols^44^. Neurons were replated into 96-well plates on day 4 and maintained in culture until day 30, when lentiviral shRNA constructs targeting each gene of interest or a non-targeting control were introduced. Lentiviral particles were generated in-house from MISSION shRNA plasmids (MilliporeSigma) as previously described^76^. Mechanical transection was performed on day 35 using the Incucyte^®^ WoundMaker Tool (Sartorius, #4563). For drug perturbation experiments, cultures were treated immediately following injury with either vehicle control or the indicated compound at the specified concentration. Neurite regeneration was quantified by daily high-content imaging for 3 days using the Thermo Scientific CellInsight CX7 LZR High-Content Screening Platform, and neurite tracing was performed using the Neuronal Profiling v4.2 application in Thermo Scientific HCS Studio Cellomics Scan v6.6.2.

### Gene expression analysis by qPCR

All molecular biology and quantitative PCR procedures were performed according to the manufacturers' protocols. On day 38, neurons were harvested, wells from each condition were pooled into five independent biological samples, and total RNA was isolated using the PicoPure RNA Isolation Kit (Thermo Fisher Scientific, #KIT0204). First-strand cDNA was synthesized using the SuperScript III First-Strand Synthesis System (Thermo Fisher Scientific, #18080051). Quantitative PCR was performed using Power SYBR^™^ Green PCR Master Mix (Thermo Fisher Scientific, #4367659) with 100 ng cDNA and 1 μM primers. Relative gene expression was quantified using the 2^−ΔΔCt method with GAPDH as the reference gene. Primer sequences were as follows: *ARHGEF12* forward, AGGAACAGACGCTGGATACCTG; reverse, GTGTGCCATCTAAGGTGTCTCC; GAPDH forward, GTCTCCTCTGACTTCAACAGCG; reverse, ACCACCCTGTTGCTGTAGCCAA (Integrated DNA Technologies).

## Quantification and statistical analysis

Statistical analyses were performed in R (v4.3.3) and Python (v3.12.9). Differential expression analysis was performed using PyDESeq2 as described above. Genes with a padj ≤ 0.05 and |log_2_fold change| ≥ 0.25 were considered significantly differentially expressed and used for all downstream functional analyses. For Ingenuity Pathway Analysis (IPA), canonical pathways with Benjamini–Hochberg false discovery rate (FDR) ≤ 0.05 were considered significantly enriched and used for downstream analyses and visualization. Diseases and biological functions and upstream regulators with enrichment p ≤ 0.05 were considered significant. For Enrichr transcription factor enrichment analysis, transcription factor terms with a padj ≤ 0.05 were considered significantly enriched.

Neurite regeneration data were analyzed in R (v4.3.3) using linear mixed-effects models implemented with the lme4 (v1.1.37) and emmeans (v2.0.3) packages. Experimental condition, imaging day, and their interaction were included as fixed effects, whereas well was modeled with random intercepts and random linear slopes over time to account for repeated measurements. Pairwise comparisons between each knockdown condition and the non-targeting control were performed at each imaging day using estimated marginal means. Unless otherwise specified, all statistical tests were two-sided, data are presented as mean ± SEM, and p-values were adjusted for multiple comparisons using FDR correction, as appropriate. Exact n values, statistical tests, and adjusted p-values are reported in the corresponding figure legends. Statistical tests, sample sizes, and significance thresholds are provided in the corresponding figure legends.
